# The effect of a manual instrumentation technique on five types of premolar root canal geometry assessed by microcomputed tomography and three-dimensional reconstruction

**DOI:** 10.1186/1471-2342-11-14

**Published:** 2011-06-15

**Authors:** Ke-Zeng Li, Yuan Gao, Ru Zhang, Tao Hu, Bin Guo

**Affiliations:** 1 State Key Laboratory of Oral Diseases, West China College of Stomatology, Sichuan University, Chengdu, P.R. China; 2 Institute of Stomatology, Chinese PLA General Hospital, Beijing, P.R. China

**Keywords:** Manual instruments, Microcomputed tomography, Root canal preparation, Root canal system, Three-dimensional imaging

## Abstract

**Background:**

Together with diagnosis and treatment planning, a good knowledge of the root canal system and its frequent variations is a necessity for successful root canal therapy. The selection of instrumentation techniques for variants in internal anatomy of teeth has significant effects on the shaping ability and cleaning effectiveness. The aim of this study was to reveal the differences made by including variations in the internal anatomy of premolars into the study protocol for investigation of a single instrumentation technique (hand ProTaper instruments) assessed by microcomputed tomography and three-dimensional reconstruction.

**Methods:**

Five single-root premolars, whose root canal systems were classified into one of five types, were scanned with micro-CT before and after preparation with a hand ProTaper instrument. Instrumentation characteristics were measured quantitatively in 3-D using a customized application framework based on MeVisLab. Numeric values were obtained for canal surface area, volume, volume changes, percentage of untouched surface, dentin wall thickness, and the thickness of dentin removed. Preparation errors were also evaluated using a color-coded reconstruction.

**Results:**

Canal volumes and surface areas were increased after instrumentation. Prepared canals of all five types were straightened, with transportation toward the inner aspects of S-shaped or multiple curves. However, a ledge was formed at the apical third curve of the type II canal system and a wide range in the percentage of unchanged canal surfaces (27.4-83.0%) was recorded. The dentin walls were more than 0.3 mm thick except in a 1 mm zone from the apical surface and the hazardous area of the type II canal system after preparation with an F3 instrument.

**Conclusions:**

The 3-D color-coded images showed different morphological changes in the five types of root canal systems shaped with the same hand instrumentation technique. Premolars are among the most complex teeth for root canal treatment and instrumentation techniques for the root canal systems of premolars should be selected individually depending on the 3-D canal configuration of each tooth. Further study is needed to demonstrate the differences made by including variations in the internal anatomy of teeth into the study protocol of clinical RCT for identifying the best preparation technique.

## Background

The study of dental and root canal morphology is a critical theme in endodontic education, training, and treatment [1-5]. Together with diagnosis and treatment planning, a good knowledge of the root canal system and its frequent variations is an absolute necessity for successful root canal therapy [6]. Hess (1921) reported on the wide variation and complexity of root canal systems, establishing that a root with a tapering canal and a single foramen was the exception rather than the rule [7]. Weine (2004) categorized the canal systems in any one root into types I-IV [8] and Yoshioka et al. [9] (2004) added type V to Weine's classification. Vertucci (2005) described a much more complex canal system and identified eight different pulp space configurations [4].

Among these classifications, Weine's classification does not consider the possible positions for large auxiliary canals or the position at which the apical foramina exit the root. Any combination of these factors could be present in any dimension of the canals, regardless of any specific configuration. Thus, Weine's classification uses a simple, direct, and clinically oriented approach [8].

In addition, a number of conventional techniques have been used for evaluating root canal morphology and the shaping ability and cleaning effectiveness of various instruments [5,10-14]. Using the modified muffle system, the exposure of radiographs under reproducible conditions in two directions (buccolingual and mesiodistal) was guaranteed to take radiographs before, during and after root canal preparation [14]. Tooth decalcification allowed effective histological evaluation of the preparation [13], but the destruction of the specimens by the muffle system and decalcification may impede the simultaneous investigation of different parameters of root canal preparation. In addition, periapical radiographs provide only two-dimensional information about root canal morphology [12].

Recently, microcomputed tomography (micro-CT) has emerged as a powerful tool for the evaluation of root canal morphology [15]. Micro-CT technology allows noninvasive evaluation of both the external and internal morphology of a tooth in a detailed and accurate manner [2,3]. Though micro-CT is expensive and time-consuming and not suitable for clinical use, it would be an effective way to examine the shape of the root canal after preparation [16,17] and obturation [18]. In comparison, the cone-beam computed tomography (CBCT) designed for dental use can provide the clinician with an imaging modality that is capable of providing a 3D representation of the maxillofacial region with minimal distortion, and it can also enhance detection and mapping the root canal system with the potential to improve the quality of root canal therapy [5,19,20].

Preparation of root canal systems includes both enlargement and shaping of the complex endodontic space together with its disinfection. A variety of instruments and techniques have been developed and described for this critical stage of root canal treatment [10]. Studies of the efficacy of various instruments for root canal preparation have typically been performed in simulated canals with simple anatomy [21,22] or in extracted human teeth with different curvatures [5,10,12-14,23,24].

Nickel-titanium (NiTi) rotary instruments were introduced to improve root canal preparation. Hand NiTi instruments can also be selected instead of rotary instruments in teeth with difficult canal anatomy and/or problematic handpiece access [25]. The ProTaper for hand use (HPT) appeared as an alternative NiTi instrument to the rotary ProTaper, embodying the same philosophy, indications, and sequence, but at a lower cost. The instrumentation is entirely manual, dispensing with the use of an electric motor [26]. The HPT instruments are recommended for use in reaming or "modified balanced forces" motion, differing from the motor-driven NiTi instruments [27]. Some limited studies of HPT instruments have been carried out [26-28]. An evaluation of preparation efficacy that integrates the canal system classification with various instruments has not been performed.

Therefore, the object of this study was to demonstrate the difference made by including variations in internal anatomy of premolars into the study protocol for investigation of a single instrumentation technique (HPT) using micro-CT and 3-D reconstruction. This is an observational report with limited numbers of teeth and techniques. And further study with large sample size is needed to obtain the clinically relevant conclusions.

## Methods

### Specimen selection and preparation

Thirty single-root premolars were randomly selected from a collection of extracted human teeth from a Chinese population sample based on mature apices without visible apical resorption and no prior endodontic treatment. These teeth were extracted because of periodontitis or orthodontic need. After understanding and written consent was obtained from patients, the extracted teeth were collected by the West China Hospital of Stomatology for teaching and research. The present study was approved by the Ethics Committee of the West China Hospital of Stomatology, and the premolars were selected from the teeth bank of the hospital. After extraction, tissue fragments and calcified debris were removed from the teeth by scaling and the teeth were stored in 0.1% thymol until used.

Preoperative radiographs of each selected tooth were first taken from the buccolingual and mesiodistal directions, then their canal system classifications were evaluated by an endodontist. After the radiographic evaluation and without probing the canals for patency so as to avoid modifying the canals' apical anatomy, premolars whose canal systems were classified into one of four categories (types I, II, III, IV) according to Weine's classification [8] were scanned using a micro-CT system (μCT-80; Scanco Medical, Bassersdorf, Switzerland) with an isotropic voxel size of 36 μm. Images were acquired from 622 slices of each tooth. From these images, a 3-D model of the tooth and canal system was constructed with a framework system (MeVisLab 2.0, MeVis Research, Bremen, Germany) on a personal computer (Athlon II X2, 2.8 GHz CPU, 2 Gbyte RAM, Windows XP) according to a customized application framework using MeVisLab [29]. The reconstructed 3-D model of the root canal system in each premolar was carefully examined. Unexpectedly, in addition to the four categories of Weine's classification [8], we found one mandibular first premolar whose canal system was classified as type V based on the system of Yoshioka *et al. *[9], which was not recognized by the professional endodontist from the preoperative periapical radiographs. Finally, five representative single-root premolars (teeth A, B, C, D, and E), each of whose canal system was classified into one of five categories (types I, II, III, IV, and V), were included in this study. In addition to the reconstruction of the tooth and canal, we also calculated the root canal diameter and showed its morphology with a 3-D color-coded image of the prepreparation canal systems.

After preparing a standard access cavity with #2 and #4 high-speed round carbide burs, using ample water cooling, each root canal was passively negotiated with a #10 K-file to the apical foramen. The working length of the canal was determined by observing the tip of the file protruding through the apical foramen and subtracting 1 mm from the recorded length.

In tooth E, however, the entrance of the lingual canal formed a perpendicular angle with the buccal canal, so we could not directly explore this canal with a #10 K-file. To find the lingual canal orifice, we adjusted the procedure: based on the length and location of the canal identified through the 3-D tooth model, a #40 K-file was used to remove the overhanging dentin above the orifice of the lingual canal, filing toward the occlusal surface. Chelating agents were also used to soften the overhanging dentin during this procedure. After the overhanging dentin was cleared, we explored the lingual canal with #8 and #10 K-files, then the working length was determined as per the aforementioned method.

According to the manufacturer's instructions, all root canals were explored with a #15 K-file after exploring with a #10 K-file, then the canals were prepared using a set of new HPT instruments (Dentsply Maillefer). Instrumental sequences followed the manufacturer's instructions: first, canals were flared coronally with S1 (followed by SX if necessary), then their working lengths were measured and confirmed with a #15 K-file, after which they were prepared with S1, S2, F1, F2, and F3 at the working length, using a "modified balanced forces" motion.

During preparation, RC-Prep (Premier Products, Plymouth Meeting, PA) was used as the lubricant. Irrigation was performed with 2 ml of 1% sodium hypochlorite (NaOCl) solution after each instrument and canal patency was ascertained with a #10 K-file for each canal. All root canals were prepared by a single operator.

### Micro-CT measurement and 3-D evaluation

After preparation with each HPT instrument at the working length, canals were dried with sterile paper points, then the teeth were scanned using the same micro-CT system. A series of micro-CT images were again obtained with the same isotropic voxel size of 36 μm.

To evaluate the efficacy of root canal preparation, volumes of interest were selected extending from the cemento-enamel junction to the apex of the roots. Using the customized application framework MeVisLab [29], the canal models (pre- and postpreparation) were reconstructed and superimposed and the instrumentation characteristics were quantitatively measured in 3-D. Numeric values were obtained for canal surface area, volume, volume changes, percentage of untouched surface, dentin wall thickness, and the thickness of dentin removed. The location of dentin removed or a lack of change during preparation were also demonstrated using the 3-D color images. Preparation errors such as canal straightening, ledging, elbow formation, or zipping were also evaluated using the color-coded reconstruction.

## Results

### Noninstrumented specimens

**Figure 1:**
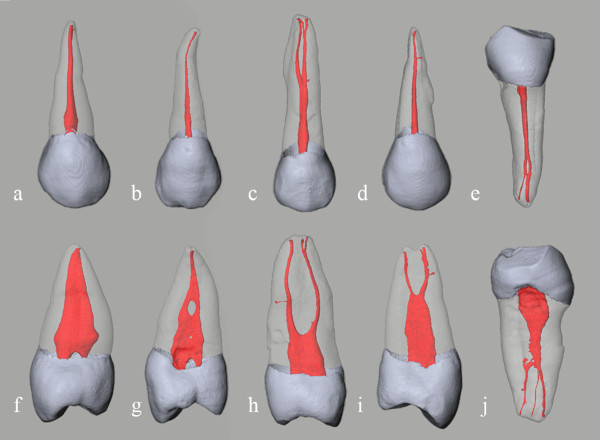
**3-D image shows the enamel, dentin, and root canal with surface rendering**. Root canal system classification: (a, f) type I of Tooth A, (b, g) type II of Tooth B, (c, h) type III of Tooth C, (d, i) type IV of Tooth D, (e, j) type V of Tooth E. Except for mandibular premolar E, teeth were maxillary premolars. The images in the top and bottom row are viewed from buccolingual and mesiodistal directions, respectively.

**Figure 2:**
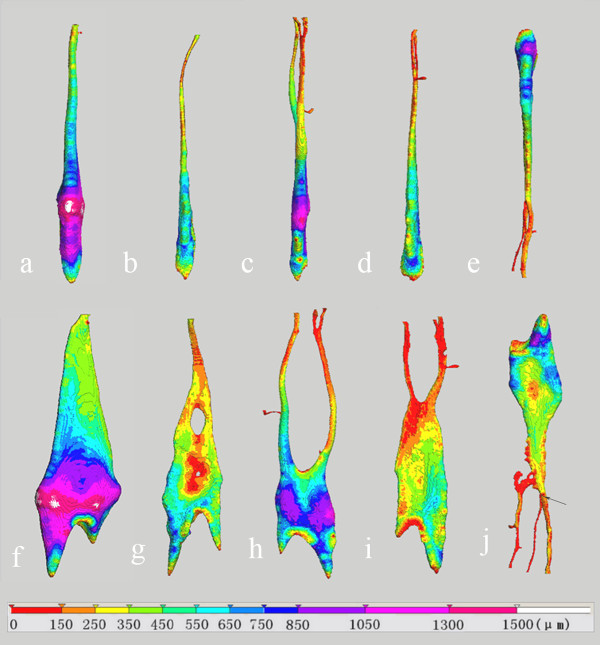
**3-D color-coded image shows the canal diameter distribution of prepreparation canals**. The bar indicates the diameter expressed in μm. The images in the top and bottom row are viewed from buccolingual and mesiodistal directions, respectively. Letters indicate the same teeth as in Figure 1. The arrow indicates the entrance of the additional canal of Tooth E.

For the five single root premolars whose root canal systems were classified into one of five types, volume rendering revealed detailed 3-D images of the root canal system, dentin, and enamel (Figure 1) and the various curves of the root canal were also shown in the 3-D model of the prepreparation canal (Figure 1, 2).

The 3-D-coded images of the canal diameter and its distribution are shown in Figure 2. The canal diameter of the main root canals of all teeth is greater than 100 μm, except that the entrance of the additional canal in Tooth E is less than 60 μm.

### Effect after instrumentation

**Figure 3:**
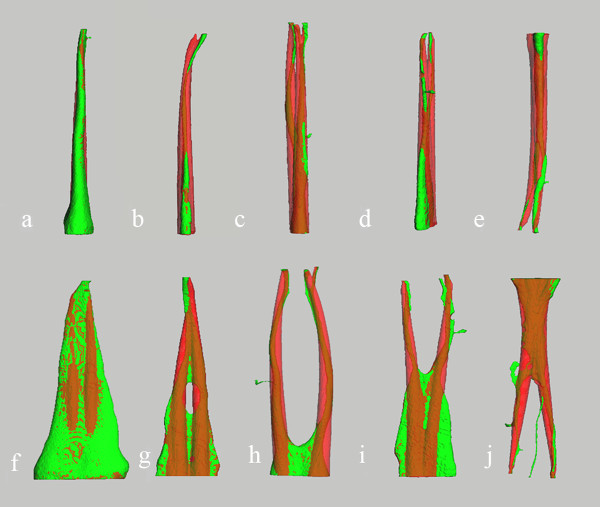
**3-D color compound images showing the pre- and postpreparation effects**. Prepreparation canal systems (green); the change in canal shapes post preparation (red). Mixed colors indicate superposition; green color alone shows the surfaces untouched during shaping. The images in the top and bottom row are viewed from buccolingual and mesiodistal directions, respectively. Letters indicate the same teeth as in Figure 1.

The gross canal anatomy of all teeth was substantially changed after root canal preparation with F3 (Figure 3). A gradual increase in the diameter along the length of the canal was noted. The canal volumes and surface areas were increased after instrumentation in all teeth. Superposition images of noninstrumented and instrumented canals reveal a wide range (27.4-83.0%) in the proportion of surfaces unchanged during preparation. Table 1 shows the increases in canal volume (Δ*V *in mm^3^) and surface area (Δ*A *in mm^2^) and the percentages of unchanged surface (Δ*P*) for the five types canal systems. The type I canal system of Tooth A showed the least increases of canal volume and surface area (less than 5%) and largest unchanged surface (83%). The type II canal system of Tooth B and the type V canal system of Tooth E revealed the highest increases of canal volume and surface area (more than 146%), and least unchanged surface (less than 29%), and the additional canal of Tooth E remained untouched. In addition, the type III canal system of Tooth C and the type IV canal system of Tooth D had the middle increases of canal volume, surface area, and unchanged surface.

**Table 1:** Changes in canal volumes, surface areas, and the percentages of surface unchanged after preparation

	Tooth (canal system classification)				
	
	A (type I)	B (type II)	C (type III)	D (type IV)	E (type V)
Δ Volume (mm^3^) *	0.84	5.67	7.30	4.05	4.16
	+4.8%	+150.2%	+96.7%	+85.6%	+146.3%
Δ Area (mm^2^) *	2.95	19.99	28.26	15.59	17.57
	+3.4%	+42.9%	+42.4%	+32.9%	+49.0%
Δ Percentage (%)	83.0%	28.9%	41.1%	40.7%	27.4%

*Absolute scores (upper lines) and relative findings (lower lines), expressed as percentages of the initial values.

**Figure 4:**
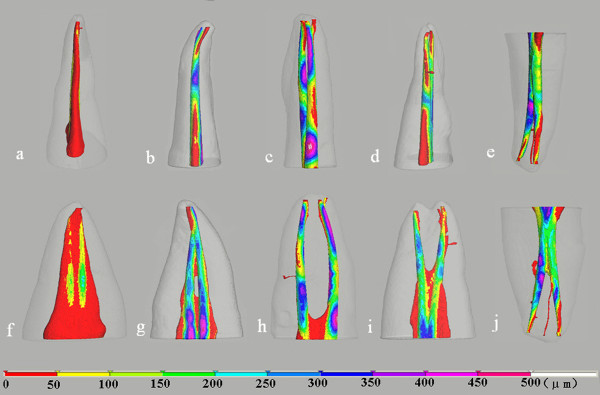
**Color-coded distance images showing the changes in canal shape during instrumentation**. The distance also indicates the amount of dentin removed. The images in the top and bottom row are viewed from buccolingual and mesiodistal directions, respectively. Letters indicate the same teeth as in Figure 1.

In the mesiodistal direction, all main canals were straightened after preparation with an F3 instrument and the straightening tended to occur toward the inner aspects of the curved parts of the root canal of teeth B, C, D and E (Figure 3). However, when viewed from the buccolingual direction, there was a transportation toward the outer aspects of the root canal and ledge formation at the apical third curve of Tooth B. Corresponding with the trend in canal transportation, more dentin was removed at the inner aspects of the curved parts and the outer aspect at the apical third curve of Tooth B (Figure 4).

**Figure 5:**
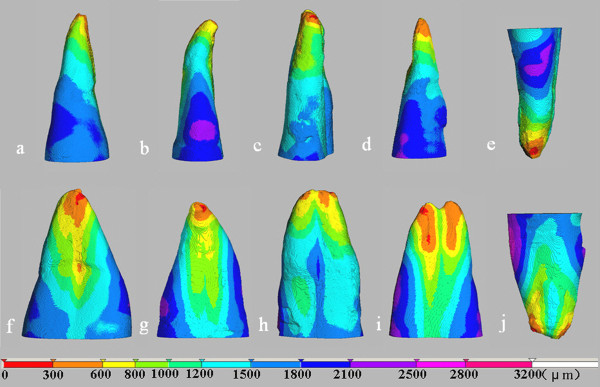
**Color-coded images showing the distribution of thickness between the root external surface and canal surface**. The images in the top and bottom row are viewed from buccolingual and mesiodistal directions, respectively. Letters indicate the same teeth as in Figure 1.

The dentin wall thicknesses of canals after they were prepared with an F3 instrument are presented in Figure 5. Except for the mesial side of the lingual canal of Tooth D, all the dentin wall thicknesses were more than 0.3 mm. However, in a 1 mm zone near the apical foramen most of the dentin wall thicknesses were less than 0.3 mm. A hazardous zone was noted that was caused by the ledge formation in tooth B. No other preparation error or HPT instrument fractures occurred during the preparation of any canal.

## Discussion

The present study aimed to reveal the differences made by including variations in internal anatomy of premolars into the study protocol for investigation of a single instrumentation technique (HPT) using micro-CT and 3-D reconstruction technology. Because of the diversity of clinical cases in endodontic therapy, we wanted to develop a study protocol that integrates the canal system classification with various instruments to evaluate the preparation efficacy using 3-D reconstruction techniques. As a pilot study, only five teeth of different categories of root canal configuration (one each of types I, II, III, IV and V) were selected. The results showed the difference of morphological changes in the five types of root canal systems shaped with the same hand instrumentation technique. However, because of the limited numbers of teeth and techniques, no statistical evaluation could be made concerning the instrumentation technique.

In the current study, color-coded images obtained by 3-D reconstruction gave insight into the morphological changes in different types of root canal systems shaped with the same instrumentation technique.

In tooth A, there is a single flattened and straight root canal that was classified as type I canal system. After F3 instrumentation, 83% of the canal surface still remained unchanged. A similar result was also found for the widened canal after it was prepared by rotary ProTaper instruments [17].

In the type II canal system of tooth B, there are multiple or S-shaped curves in the canals and an isthmus communication between two root canals localized in the coronal third. After it was prepared with F3, the volume of the canal system increased by 150.2% and a ledge was formed in the apical curved part. The ledge was possibly caused by the additive effects of instrumentation to the working length for both the buccal and lingual canals, but the use of chelating agents [30] and the much more complicated root canal anatomy with curves in multiple positions and planes could also have contributed [15]. When the morphology of the root canals after preparation with S1, S2, F1, and F2 were also evaluated from 3-D canal models (data not shown), we found that significant transportation was created after the use of F1 and a ledge was formed after the use of F2. These results may suggest that the working length needs to be reconfirmed after instrumentation of a type II canal system as in Tooth B using F1 [31].

Although there were multiple or S-shaped curves in the type III canal system of Tooth C and the type IV canal system of Tooth D, we achieved a good instrumentation effect in the coronal and middle third after the use of F3. However, there was an additional untouched canal surface caused by apical transportation in the apical third and we found it impossible to prepare the accessory canal in the apical third using HPT instruments, except by exploring it with small, precurved K-files. In clinical cases where the canal systems are like those in Tooth C and Tooth D, these unchanged surfaces may harbor microorganisms and allow for the presence of residual infection post treatment [32].

In the type V canal system of Tooth E, it is regrettable that we could not accomplish the instrumentation of the additional canal after preparing the other two canals with F3. The volume increased up to 146.3%, mainly caused by the removal of the dentin protuberance. In clinical cases that have type V canal systems like Tooth E, CT data and 3-D canal models could be useful to guide instrumentation and the use of a dental operating microscope and ultrasonic technique would be suitable for more efficiently exploring and finding the unusual canal access [1,33].

In the mesiodistal direction, all main canals were straightened after preparation with an F3 instrument and the straightening tended to occur toward the inner aspects of the curved parts of the root canal of teeth B, C, D and E (Figure 3). Our results are consistent with those of Yang [34]. In previous studies [35,36], the ProTaper Universal instruments with noncutting tips showed better performance than the conventional ProTaper instruments for root canal transportation. However, Özer reported that all three rotary systems (ProTaper Universal, Hero 642 Apical, FlexMaster) showed similar results during preparation of curved root canals and for transportation despite their noncutting tips [37].

For the five types of canal systems in this study, there was a wide range (27.4-83.0%) in the percentage of surface unchanged during preparation. The untouched area was distributed in the recesses of the flattened canal, the isthmus of the type II canal system, the outer aspects of multiple or S-shaped curves, lateral and accessory canals, and the additional canal of Tooth E. It has been shown that frequent and copious irrigation with sodium hypochlorite not only flushes out debris from the canal lumen, but also dissolves organic tissue in the noninstrumented areas and the predentin layer [38]. More dentin debris can be removed from the isthmus, oval extensions in the root canal, and irregularities of the root canal wall by the use of ultrasonic irrigation with NaOCl as the irrigant [39,40]. Furthermore, in the mid-1990s, a method and device was presented that allowed cleansing of root canals without the need for manual instrumentation, and this noninstrumental hydrodynamic technique (NIT) showed an equal or even better cleanliness in all root sections than hand instrumentation [41,42]. Thus, the untouched area within each type of canal system configuration, although not amenable to mechanical debridement, might be cleaned by these means.

In daily clinical practice, there are some cases in which conventional intraoral radiography and/or panoramic radiography alone do not provide enough information on the pathologic condition [43]. It is important to visualize and to have knowledge of the internal tooth anatomy before undertaking endodontic therapy [4]. Micro-CT has emerged as a powerful tool for evaluation of root canal morphology. Unfortunately, this technique is not suitable for clinical use, but cone-beam computed tomography (CBCT) systems have now been introduced for 3-D imaging of hard tissues of the maxillofacial region, with minimal distortion [44]. When encountering a case with a complicated canal system (such as type II or V) by radiographic evaluation, CBCT may be a good choice to allow appropriate management of the endodontic problem for endodontists [19,44].

## Conclusions

From 3-D color-coded images, we discovered obviously different morphological changes in the five types of root canal systems shaped with the same hand instrumentation technique. These results provide further information that premolars are among the most difficult teeth to be treated endodontically and that instrumentation techniques for the root canal systems of premolars should be judged individually depending on the 3-D canal configuration of each tooth. Further study is needed to demonstrate the differences made by including variations in internal anatomy of teeth into the study protocol for investigation of various instrumentation techniques.

## Competing interests

The authors declare that they have no competing interests.

## Authors' contributions

KZL, YG, RZ, TH and BG participated in the design of the experiment and wrote the manuscript. KZL and YG participated in the acquisition, analysis and interpretation of data. All authors read and approved the final manuscript.

## Pre-publication history

The pre-publication history for this paper can be accessed here:

http://www.biomedcentral.com/1471-2342/11/14/prepub
